# Histopathological Analysis Post Sleeve Gastrectomy: Value and Correlation With Preoperative Endoscopic Findings

**DOI:** 10.7759/cureus.78853

**Published:** 2025-02-11

**Authors:** Bandar Saad Assakran, Abdulaziz S Al-lihimy, Sarah A Alkuraydis, Aseel M Alsamaani, Ghaida S Alabdulaaly, Deema Khalid Alshaya, Ola E Alkhoshiban

**Affiliations:** 1 General Surgery, King Fahad Specialist Hospital, Buraydah, SAU; 2 General Surgery, Buraydah Central Hospital, Buraydah, SAU; 3 College of Medicine, Qassim University, Buraydah, SAU; 4 Unaizah College of Medicine and Medical Sciences, Qassim University, Unaizah, SAU

**Keywords:** bariatric surgeries, cost-effectiveness analysis, histopathological examination, post-sleeve gastrectomy, pre-operative endoscopic findings

## Abstract

Background

Laparoscopic sleeve gastrectomy (LSG) is one of the most common bariatric surgical procedures performed today. Before LSG, patients undergo an endoscopic examination to assess for any significant pathology that could affect the surgical outcome. Following LSG, the resected stomach tissue is routinely submitted for histopathological analysis, though the practice's efficacy remains debated. Furthermore, formal guidelines and recommendations for this practice are lacking.

Methodology

This retrospective single-center analysis was conducted at King Fahad Specialist Hospital (KFSH) in Buraydah, Al-Qassim. Following ethical approval, all patients with obesity who underwent LSG at our institution between 2017 and 2024 and whose medical records contained complete data were included in this study. Patient data meeting the inclusion criteria were then collected through a review of medical records. Data were collected using a standardized collection sheet.

Results

This study included 908 patients with obesity. Of the 908 patients, 547 (60.2%) were female and 361 (39.8%) were male. The vast majority, 893 (98.3%), were Saudi nationals. Participants had a mean age of 35.16 ± 13.049 years and a mean BMI of 44.08 ± 7.095 kg/m^2^. Preoperative gastroscopy revealed no mucosal abnormalities, ulcers, polyps, or vascular lesions in 421 patients (99.3%). Histopathological examination (HPE) of the resected tissue identified premalignant lesions in three patients (0.7%), with no malignancies detected.

Conclusions

This study highlights the value of HPE and its correlation with preoperative endoscopic findings in patients undergoing LSG. Given the low prevalence of malignancy, routine HPE may not be warranted. Therefore, HPE should be considered selectively to reduce costs and effort.

## Introduction

Laparoscopic sleeve gastrectomy (LSG) is a bariatric surgical procedure that facilitates weight loss and addresses obesity-related comorbidities [1]. As the global prevalence of obesity rises, so does the bariatric surgery rate [2]. Globally, over 685,000 bariatric surgeries were performed recently [3]. LSG accounts for over half of all bariatric surgeries around the world [3]. Sleeve gastrectomy has become the most common and preferred bariatric surgery among both patients and doctors [1,4,5]. The procedure's relative simplicity and effectiveness in achieving weight loss contribute to its increasing popularity [1,5].

While sleeve gastrectomy offers significant health benefits, potential complications include malnutrition, cholelithiasis, gastroesophageal reflux disease, and leaks [6].

Esophagogastroduodenoscopy (EGD) is recommended for all bariatric surgical procedures. EGD offers several important benefits, including the detection and diagnosis of premalignant and malignant lesions in the upper gastrointestinal tract. This facilitates timely intervention and treatment, which significantly improves patient prognosis and long-term health outcomes [7]. Despite some surgeons' reluctance to perform routine upper endoscopy preoperatively, a study revealed its clinical significance: endoscopic findings led to the cancellation or postponement of 63.8% of procedures [8]. Schlottmann et al. reported that 29.4% of patients experiencing no symptoms had an abnormality in EGD [9].

Following LSG, the resected stomach tissue undergoes routine histopathological examination (HPE). While the clinical significance of routine HPE of resected stomach tissue following LSG remains debated, studies have shown unpredictable microscopic changes in these samples. These changes can include intestinal metaplasia, *Helicobacter pylori* infection, and gastrointestinal stromal tumors [10]. Another study revealed abnormal histopathological findings in 21.9% of cases, most commonly non-specific chronic gastritis. Significant pathological findings were present in 15% of patients, potentially requiring further workup or management [11]. Therefore, this study investigates the importance of histopathological analysis of the resected stomach after LSG.

## Materials and methods

Objective

This study aims to investigate the value of histopathological analysis of postoperative LSG and its correlation with preoperative endoscopic findings.

Methodology

This retrospective, single-center observational study was conducted from 2017 to 2024 at King Fahad Specialist Hospital (KFSH) in Buraydah, Al-Qassim region, Saudi Arabia. This study included all patients who underwent LSG at our institution from 2017 to 2024. This study included 1,610 patients who underwent LSG. Of the total sample reduced due to missing data, 908 patients with complete medical records were included.

Inclusion Criteria

This study included 908 patients with complete medical records who underwent LSG at KFSH in Buraydah, Al-Qassim, between 2017 and 2024.

Exclusion Criteria

Patients with incomplete medical records or those deemed unfit for surgery or anesthesia were excluded.

Data Collection Method

Data were collected through a review of medical records. A collection sheet was utilized to collect the data. The study collected patient demographics (age, gender, nationality, BMI), endoscopic findings (hiatal hernia, polyps, ulcer, mass, vascular lesion), and histopathological results (atrophic gastritis, intestinal metaplasia, dysplasia, gastric cancer).

Statistical analysis

Statistical data analysis was performed using SPSS (IBM Corp., Armonk, NY). Categorical or qualitative data were presented as frequencies and percentages, while quantitative or numerical data were expressed as mean ± SD, and a normality test was performed. Two-sample t-tests and ANOVA tests were used to analyze normally distributed continuous variables, while chi-square tests were used for categorical variables. Statistical significance was defined as a p-value of < 0.05.

Privacy and ethical considerations

Data collection followed ethical approval from the Al-Qassim Province Regional Research Ethics Committee (approval number: 607/46/3790). This study did not require patient consent because no personally identifiable information was collected.

## Results

The study included 908 patients with obesity. Of the 908 patients, 547 (60.2%) were female and 361 (39.8%) were male. The vast majority, 893 (98.3%), were Saudi nationals. The patients' mean age was 35.16 ± 13.049 years, and their mean BMI was 44.08 ± 7.095 (Table 1).

**Table 1:** Patient demographics (N = 908). Demographic information is presented in frequencies (n) and proportion (%).

Variables	Category	n (%)
Gender	Female	547 (60.2%)
	Male	361 (39.8%)
Nationality	Saudi	893 (98.3%)
	Non-Saudi	15 (1.7%)
Patient’s age, years	Age (mean ± SD)	35.16 ± 13.049
Patient’s BMI, kg/m^2^	BMI (mean ± SD)	44.08 ± 7.095

Endoscopic findings (Table 2) indicated that 108 patients (11.9%) had a hiatal hernia. Normal mucosa was observed in 486 patients (53.5%), while abnormal mucosa was found in 422 patients (46.5%). Polyps were observed in 15 patients (1.7%) and ulcers in 13 (1.4%). A mass was found in one patient (0.1%), and vascular lesions were present in five (0.6%).

**Table 2:** Endoscopic findings. Endoscopic findings are presented as frequencies (n) and percentages (%).

Variables	Category	n (%)
Hiatal hernia	Yes	108 (11.9%)
	No	800 (88.1%)
Mucosa	Normal	486 (53.5%)
	Abnormal	422 (46.5%)
Polyp	Yes	15 (1.7%)
	No	893 (98.3%)
Ulcer	Yes	13 (1.4%)
	No	895 (98.6%)
Mass	Yes	1 (0.1%)
	No	907 (99.9%)
Vascular lesion	Yes	5 (0.6%)
	No	903 (99.4%)

Histopathologic findings (Table 3) revealed atrophic gastritis in six patients (0.7%) and intestinal metaplasia in four (0.4%). Dysplasia and gastric cancer were not observed in any tissue samples.

**Table 3:** Histopathology results. Histopathology results are presented in frequencies (n) and proportion (%).

Variables	Category	n (%)
Atrophic gastritis	Yes	6 (0.7%)
	No	902 (99.3%)
Intestinal metaplasia	Yes	4 (0.4%)
	No	904 (99.6%)
Dysplasia	Yes	0 (0.0%)
	No	908 (100.0%)
Gastric cancer	Yes	0 (0.0%)
	No	908 (100.0%)

The relationship between patient age (years) and BMI (kg/m²) is presented in Table 4. No significant correlation was observed between age and BMI (r = -0.016, P = 0.641).

**Table 4:** Correlations. Relationship between patient age (years) and BMI (kg/m2). Pearson correlation coefficient, r. * Significant at p < 0.05.

Variables	Correlation	Patient’s age	Patient’s BMI
Patient’s age	Pearson correlation	1	-0.016
	Sig. (2-tailed), N	P < 0.001^*^, 908	P = 0.641, 907
Patient’s BMI	Pearson correlation	-0.016	1
	Sig. (2-tailed), N	P = 0.641, 907	P = 0.641, 907

Table 5 presents the chi-square test results assessing the association between endoscopic and histopathologic findings. No significant association was found between endoscopic and histopathologic findings (p = 0.419).

**Table 5:** Chi-square test. Endoscopic and histopathologic findings are presented as frequencies (n) and percentages (%). * Significant at p < 0.05.

Endoscopic findings	Histopathological findings		P-value
	Normal	Abnormal	
Normal	421 (99.3%)	3 (0.7%)	0.419
Abnormal	478 (98.8%)	6 (1.2%)	

Patient characteristics stratified by endoscopic findings are presented in Table 6. Male patients exhibited a significantly higher prevalence of abnormal endoscopic findings than female patients (p < 0.001). No significant associations were found between endoscopic findings and demographic variables or histopathology.

**Table 6:** Patient attributes by endoscopic findings. Patient attributes by endoscopic findings are presented as frequencies (n) and percentages (%). * Significant at p < 0.05.

Variables		Endoscopic findings		P-value
		Normal	Abnormal	
Gender	Female	288 (52.7%)	259 (47.3%)	<0.001*
	Male	136 (37.7%)	225 (62.3%)	
Nationality	Saudi	418 (46.8%)	475 (53.2%)	0.600
	Non-Saudi	6 (40.0%)	9 (60.0%)	
BMI	BMI < 40 kg/m^2^	98 (43.8%)	126 (56.2%)	0.257
	BMI > 40 kg/m^2^	320 (48.1%)	345 (51.9%)	
Hiatal hernia	Yes	0 (0.0%)	108 (100.0%)	<0.001*
	No	424 (53.0%)	376 (47.0%)	
Mucosa	Normal	424 (87.2%)	62 (12.8%)	<0.001*
	Abnormal	0 (0.0%)	422 (100.0%)	
Polyp	Yes	0 (0.0%)	15 (100.0%)	<0.001*
	No	424 (47.5%)	469 (52.5%)	
Ulcer	Yes	0 (0.0%)	13 (100.0%)	<0.001*
	No	424 (47.4%)	471 (52.6%)	
Mass	Yes	0 (0.0%)	1 (100.0%)	0.349
	No	424 (46.7%)	483 (53.3%)	
Vascular lesion	Yes	0 (0.0%)	5 (100.0%)	0.036*
	No	424 (47.0%)	479 (53.0%)	
Atrophic gastritis	Yes	3 (50.0%)	3 (50.0%)	0.871
	No	421 (46.7%)	481 (53.3%)	
Intestinal metaplasia	Yes	1 (25.0%)	3 (75.0%)	0.383
	No	423 (46.8%)	481 (53.2%)	

Patient characteristics stratified by histopathologic findings are presented in Table 7. No statistically significant associations were observed between demographic characteristics, endoscopic findings, and histopathologic results.

**Table 7:** Patient attributes by histopathological findings. Patient attributes by histopathologic findings are presented as frequencies (n) and percentages (%). * Significant at p < 0.05.

Variables		Histopathological findings		P-value
		Normal	Abnormal	
Gender	Female	541 (98.9%)	6 (1.1%)	0.692
	Male	358 (99.2%)	3 (0.8%)	
Nationality	Saudi	884 (99.0%)	9 (1.0%)	0.696
	Non-Saudi	15 (100.0%)	0 (0.0%)	
BMI	BMI < 40 kg/m^2^	222 (99.1%)	2 (0.9%)	0.836
	BMI > 40 kg/m^2^	658 (98.9%)	7 (1.1%)	
Hiatal hernia	Yes	108 (100.0%)	0 (0.0%)	0.268
	No	791 (98.9%)	9 (1.1%)	
Mucosa	Normal	483 (99.4%)	3 (0.6%)	0.222
	Abnormal	416 (98.6%)	6 (1.4%)	
Polyp	Yes	15 (100.0%)	0 (0.0%)	0.696
	No	884 (99.0%)	9 (1.0%)	
Ulcer	Yes	13 (100.0%)	0 (0.0%)	0.716
	No	886 (99.6%)	9 (1.0%)	
Mass	Yes	1 (100.0%)	0 (0.0%)	0.920
	No	898 (99.0%)	9 (1.0%)	
Vascular lesion	Yes	5 (100.0%)	0 (0.0%)	0.822
	No	894 (99.0%)	9 (1.0%)	
Atrophic gastritis	Yes	0 (0.0%)	6 (100.0%)	<0.001*
	No	899 (99.7%)	3 (0.3%)	
Intestinal metaplasia	Yes	0 (0.0%)	4 (100.0%)	<0.001*
	No	899 (99.4%)	5 (0.6%)	

## Discussion

In our study, 53.5% of patients had normal mucosal alteration, while 46.5% had abnormal mucosa findings preoperatively. This contrasts with Abdallah et al. (2023), who found that 76.2% of cases had normal and/or abnormal mucosa that did not need surgical decisions, and 23% had findings that caused delays or changes in management [12]. Furthermore, a German study of 801 patients found that 65.7% had abnormal endoscopic findings preoperatively, with gastritis being the most common (32.1%) [13].

Atrophic gastritis was found in 0.7% of patients and intestinal metaplasia in 0.4% in our study, compared to Rashdan et al. (2022), who reported chronic gastritis in 88.3% and intestinal metaplasia in 2.2% and this variation could be explained by regional factors and different population [14]. Additionally, intestinal metaplasia was observed in 0.3% of the study population [15].

Among 908 resected stomach specimens, no dysplasia or cancer was identified. In their analysis of 546 cases over eight years, AbdullGaffar et al. [16] found no malignancies. In a retrospective review of 755 cases, Yardimci et al. [17] identified malignant lesions in four patients (0.5%). In their study of 925 patients, Canil et al. [15] identified neoplasms in 0.3%. Rashdan et al. [14] also reported that most gastric sleeve specimens sent for HPE yielded no serious findings.

In the current study, preoperative gastroscopy revealed no mucosal abnormalities, ulcers, polyps, or vascular lesions in 421 patients (99.3%). HPE identified premalignant lesions in three patients (0.7%), but no malignancies were found. Several studies have discouraged routine HPE of LSG specimens [18,19].

Finally, our preoperative endoscopic screening revealed no worrisome features or malignancies in the majority of cases, which was confirmed by HPE. Therefore, routine HPE for LSG patients is unwarranted, excluding clinically high-risk patients with concerning endoscopic or intra-operative findings. Moreover, larger prospective studies are warranted to validate these initial findings.

This study has several limitations. First, this was a retrospective cross-sectional study; therefore, further prospective research is needed to confirm these findings. Furthermore, reliance on medical records, which may be incomplete or poorly documented, limited data collection and could have affected the quality and representativeness of the data. Because this study was conducted in a specific location (Al-Qassim region, Saudi Arabia), the findings may reflect regional factors rather than universal trends. Consequently, these findings may not be generalizable to other populations or diverse ethnic groups. In addition to citing limitations, this study has different strengths such as its large sample size and its value in clarifying the clinical significance of HPE in bariatric surgery.

## Conclusions

This study highlights the value of correlating HPE with preoperative endoscopic findings in patients undergoing LSG. Given the absence of detected malignancies, we advise against routine HPE. Clinical background, preoperative endoscopic screening, and intraoperative macroscopic examination of resected specimens for abnormalities are recommended to improve patient management, prognosis, and cost-effectiveness.
